# Depth Perception and the History of Three-Dimensional Art: Who Produced the First Stereoscopic Images?

**DOI:** 10.1177/2041669516680114

**Published:** 2017-01-01

**Authors:** Kevin R. Brooks

**Affiliations:** Department of Psychology and Perception and Action Research Centre, Faculty of Human Sciences, Macquarie University, Sydney, Australia

**Keywords:** art, Chimenti, Dali, Da Vinci, depth, disparity, perception, stereopsis

## Abstract

The history of the expression of three-dimensional structure in art can be traced from the use of occlusion in Palaeolithic cave paintings, through the use of shadow in classical art, to the development of perspective during the Renaissance. However, the history of the use of stereoscopic techniques is controversial. Although the first *undisputed* stereoscopic images were presented by Wheatstone in 1838, it has been claimed that two sketches by Jacopo Chimenti da Empoli (c. 1600) can be to be fused to yield an impression of stereoscopic depth, while others suggest that Leonardo da Vinci’s Mona Lisa is the world’s first stereogram. Here, we report the first quantitative study of perceived depth in these works, in addition to more recent works by Salvador Dalí. To control for the contribution of monocular depth cues, ratings of the magnitude and coherence of depth were recorded for both stereoscopic and pseudoscopic presentations, with a genuine contribution of stereoscopic cues revealed by a difference between these scores. Although effects were clear for Wheatstone and Dalí’s images, no such effects could be found for works produced earlier. As such, we have no evidence to reject the conventional view that the first producer of stereoscopic imagery was Sir Charles Wheatstone.

## Introduction

The simulation of depth in artistic works presented on flat media has been a challenge for artists throughout history. Although the objects to be represented are invariably voluminous and are located at various distances, their representations on walls, paper, or canvas are necessarily two dimensional. Historically, artists throughout the world have attempted to imply depth using techniques that exploit the well-known monocular “pictorial” cues to depth. More recently, digital technology has made the use of binocular depth cues a legitimate possibility for artists; yet stereoscopic art is rather rare outside of the medium of cinema (although see Ferragallo (1974) and Wade (2007, 2009) for notable exceptions describing the work of Roger Ferragallo, Ludwig Wilding, and Calum Colvin, respectively). As the use of visual depth cues by artists has been covered extensively by various writers (Gillam, 2011; Kemp, 1990; Livingstone, 2002; Melcher & Cavanagh, 2011), only a brief history of their development is included here.

### The Development of the Use of Depth Cues Through Art History

#### Monocular depth cues

**Figure 1.**
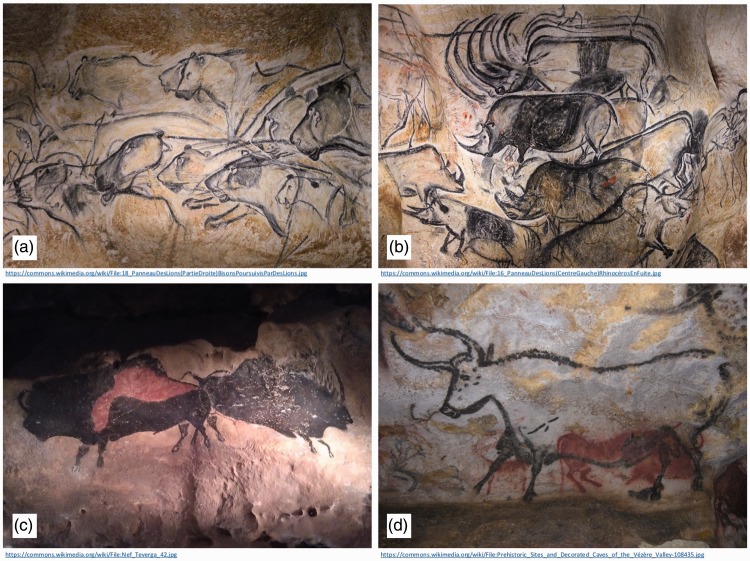
Depth cues in Palaeolithic art. (a) and (b) Occlusion and transparency in paintings in the Chauvet cave (c. 30,000 BCE). (c) and (d) Occlusion and transparency in the Lascaux cave (c.15,000 BCE).

**Figure 2.**
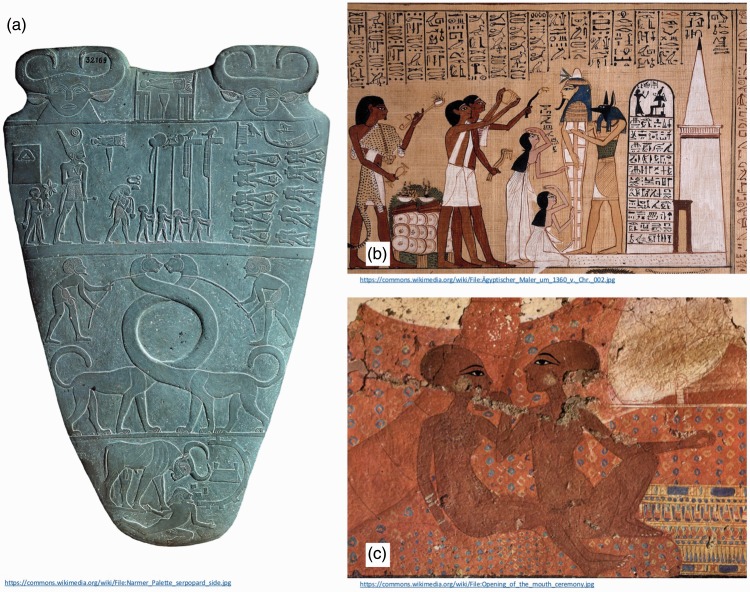
Consistent use of occlusion in ancient Egyptian art. (a) The *Palette of Narmer* and (b) *Opening of the mouth ceremony*, from the *Book of the Dead* (papyrus of Hunefer, c. 1300 BCE). (c) *Nofernoferuaton and Nofernoferure* from the Amarna period (c. 1360 BCE).

The use of occlusion is demonstrated in images in some of the very earliest examples of human art, such as the cave paintings from the Chauvet-Pont-d'Arc Cave (c. 30,000 BCE, see Figure 1(a) and (b)), or those at Lascaux, France (c. 17,000 BCE, Figure 1(c)). However, caution should be used when interpreting these (Melcher & Wade, 2006; Wade & Melcher, 2006). Many Palaeolithic images that feature occlusion also show transparency in areas where occlusion might be expected (see Figure 1(a), (b), and (d)), calling into question the extent to which Palaeolithic artists appreciated the depth cue of occlusion. As noted by Gillam (2011), failures of occlusion to effectively segregate foreground and background objects are rare in work since this time. Although ancient Egyptian works of art are often described as being rather two dimensional, lacking perspective, or the use of shadows (Binder, 2000), occlusion does feature as a dominant cue (Anderson, 2000), as seen in works such as the *Palette of Narmer* (c. 3100 BCE), *Nofernoferuaton and Nofernoferure* from the Amarna period (c. 1360 BCE), or Hunefer’s *Book of the Dead* (c. 1300 BCE), shown in Figure 2.

**Figure 3.**
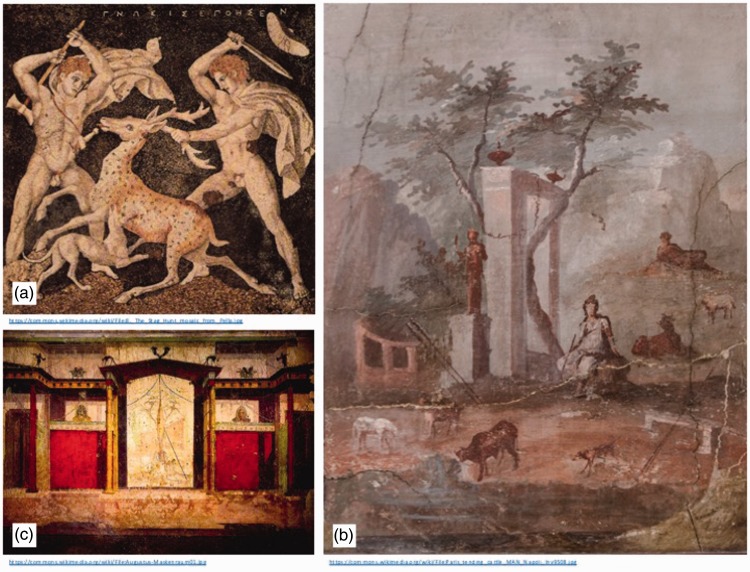
Depth cues in classical art. (a) *The Stag Hunt* mosaic by Gnosis (c. 300 BCE) demonstrates the use of attached shadow. (b) Pompeiian fresco *Paris on Mount Ida*, including the depth cues of shading gradients and aerial perspective. (c) Roman fresco in the *Room of the Masks*, House of Augustus, showing examples of the use of the cast shadow.

The use of shading gradients (often referred to as “attached shadows”: Mather, 2016) to provide an improved sense of surface curvature and volume (using the technique known as *skiagraphia*: literally “shadow painting”) was developed by Greek painter Apollodorus (c. 480 BCE). Although his works have long been lost, evidence of the use of such shading can still be seen in work such as the Macedonian mosaic known as *The Stag Hunt* (see Figure 3(a)) by Gnosis (c. 300 BCE), or the 1st century Pompeiian painting *Paris on Mount Ida* (Gombrich, 1977). Demonstrating a sophisticated understanding of the interplay of light and form that is shared by many works of the era, this fresco also shows early use of aerial perspective to communicate depth (Figure 3(b)). In addition, cast shadows were used to enhance the impression of three-dimensional (3D) structure in examples such as the *Room of the Masks* in the House of Augustus, Rome (Casati, 2003), believed to have been painted around 30 BCE (Figure 3(c)).

**Figure 4.**
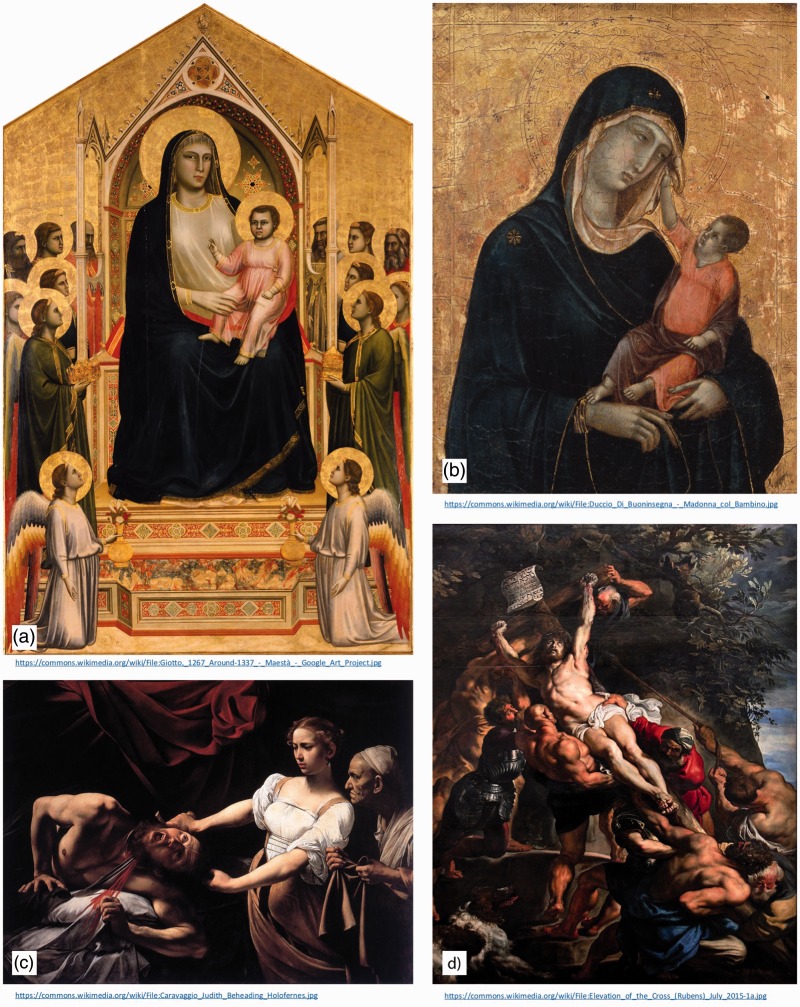
Depth cues in medieval and Renaissance art. (a) *Ognissanti Madonna*, by Giotto (c. 1310). (b) *Madonna and Child*, by Duccio (c. 1300). (c) *Judith Beheading Holofernes*, by Caravaggio (1598–1599). (d) *Elevation of the Cross*, by Peter Paul Rubens (1610–1611).

While cast shadows are often absent from artistic works since this date (Gombrich, 1995), enthusiasm for shading gradients was renewed in the late middle ages (c. 1300) and can be seen in paintings such as those by Giotto (Figure 4(a)) and Duccio (Figure 4(b)). Later, Renaissance painters such as Caravaggio (Figure 4(c)) and Peter Paul Rubens (Figure 4(d)) employed the technique of *chiaroscuro*, using even more dramatic patterns of light and dark to imply depth.

**Figure 5.**
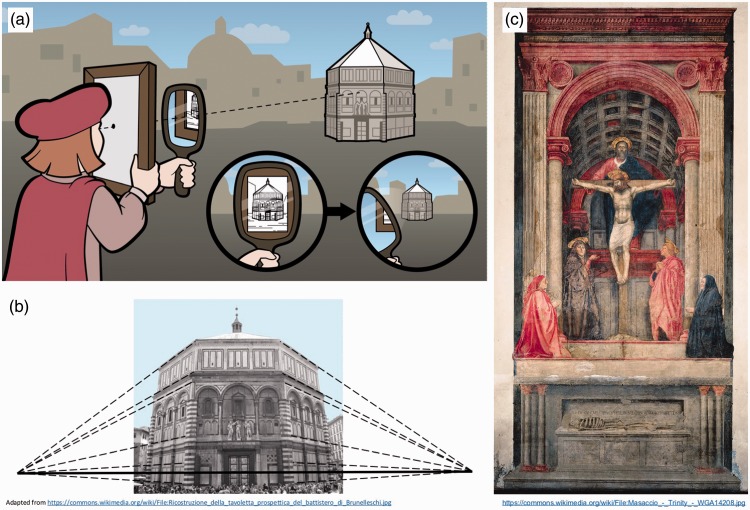
A new perspective. (a) Brunelleschi’s experiment, confirming the correspondence between the visual scene and his own perspective drawing, with the use of a (removable) mirror and a small aperture in the picture, which faced away from the observer. (b) Brunelleschi’s view of the Florence Baptistery, demonstrating 2-point perspective. (c) One of the first known coherent perspective images: Masaccio’s *The Holy Trinity*.

Early in the 15th century, the formalisation of the rules of linear perspective constituted something of a revolution in the art world, although the identity of the originator is controversial. One common story involves Italian engineer Filippo Brunelleschi who is purported to have conducted perspective experiments between 1401 and 1425 (Edgerton, 2006; Kubovy, 1986; Vasari, 1965). Brunelleschi’s empirical observations (e.g., concerning the convergence of each set of parallel lines in the image to a unique vanishing point, as in Figure 5(b)) led to predictions in the form of two “perspective panels,” one depicting the Florentine Baptistery, that should correspond to the scene as viewed from the same vantage point. These predictions were confirmed by viewing the Baptistery through a small sighting aperture in the drawing, which faced away from the artist. The interjection of a mirror into the line of sight allowed the viewer to alternate between the scene and the drawing, which were found to correspond closely (Figure 5(a) and (b)). Unfortunately, Brunelleschi’s perspective panels have long been lost. While few dispute the story of the “peephole and mirror” experiments, and that Brunelleschi appreciated the diminution of spatial extents with distance, his appreciation of vanishing points is not universally accepted (Damisch, 1994; Field, 1997). However, it is far less controversial that the first to explicitly record the rules of perspective was Leon Battista Alberti in 1435 (Alberti, 1972; Wilde, 1994) as his *costruzione legittima*. While convergent perspective can be seen in some works of art that predate Brunelleschi’s experiments (e.g., the Room of the Masks in Figure 3(c); c. 30 BCE); *Pageant of Orestes* mural, Pompeii (2nd century CE); *Jesus Before Caiaphas* (1305) by Giotto; *Presentation of Jesus at the Temple* (1342); *The Annunciation* [1344] by Lorenzetti), it was inconsistently applied, with real-world parallel lines failing to converge to a single vanishing point (Kemp, 1990; Little, 1971; Tyler, 2000; Wilde, 1994). The earliest known image with a coherent vanishing point is often credited as *The Holy Trinity*, painted Masaccio (a friend of Brunelleschi’s) in 1425 (Howard, 2012; Kubovy, 1986, although see also Field, 1997), while others credit another Italian contemporary Masolino, for examples, such as *Founding of Santa Maria Maggiore* (1423–1425) and *Healing of the Cripple and Raising of Tabitha* (1426–1427).

**Figure 6.**
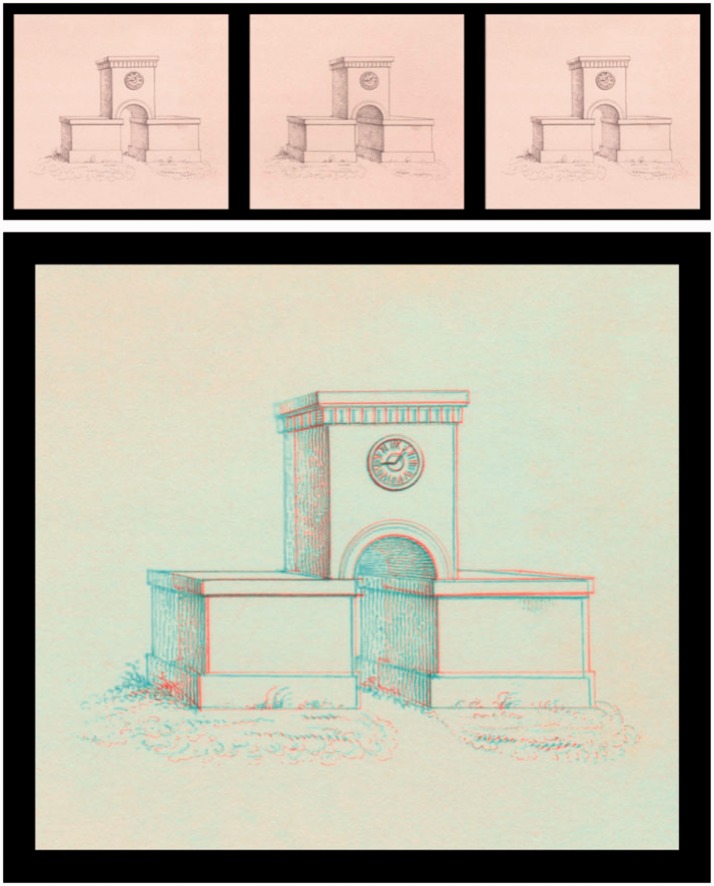
One of the first confirmed stereoscopic images: *The Wheatstone Arch* (Wheatstone, 1838). Presented in Universal Freeview (L-R-L) format (such that parallel fusion results in a stereoscopic image on the left and a pseudoscopic image on the right, while crossed fusion produces the converse) at the top, and Dubois anaglyph format below.

**Figure 7.**
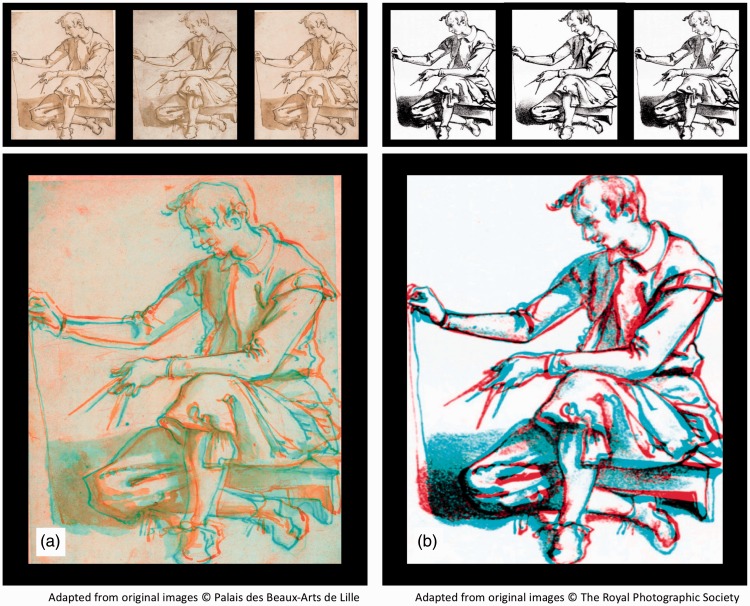
Chimenti’s stereograms, presented in Universal Freeview (L-R-L) format at the top, and Dubois anaglyph format below. (a) Original ink-wash sketches produced around 1600 by Jacopo da Empoli. (b) Woodcut facsimiles produced from copies of the drawings in 1862 (Wade, 2003). Note that as these images were completed on separate sheets, it is not known which, if any, is intended to represent the left or the right eye’s half-image.

#### Binocular depth cues

The depth cue of binocular disparity was formally introduced in 1838 by Wheatstone, who not only propounded his theory of binocular depth perception but also produced a stereoscope and several relatively simple line drawings that could be mounted therein to produce a convincing depth effect. Although these line drawings (such as the *Wheatstone Arch* example reproduced in Figure (6) represent the first *undisputed* stereograms, there are two significant claims that artists had produced stereoscopic images long before Wheatstone. These claims concern two Italian artists: Jacopo “Chimenti” da Empoli (Brewster, 1860) and Leonardo da Vinci (Carbon & Hesslinger, 2013).

Jacopo da Empoli (1551–1640), also known as Jacopo Chimenti, was an Italian artist who was born and worked principally in Florence (Porter, 1995). While he is renowned in the art world as a painter, his relevance to vision science and particularly the perception of depth lies in a pair of ink-wash sketches of the same subject, which were completed on two separate sheets around 1600 (see Figure 6(a)). Sir David Brewster suggested that the small differences between the two sketches were consistent with binocular disparities, and that when viewed appropriately, the images produced a compelling impression of stereoscopic depth (Brewster, 1860). As Brewster did not have access to the images in question (Joy, 1864; Wade, 2003), he relied on the testimony of one observer who had viewed the images (using a cross-eyed free fusion technique) in the *Musée Wicar* in Lille, France (collection now merged with the Palais des Beaux-Arts de Lille, where the images reside to this day). Many of the further observations of Brewster (and other contributors) were instead based on a pair of woodcut copies that had been made from photographs of the original images (see Figure 6(b)). While Brewster was adamant about the stereoscopic effects that these images produced for him and for all other observers to whom he had shown them, more sceptical observers remained equally adamant that they provide no such effects. This discrepancy between the reports of the believers and nonbelievers may lead to concerns of demand characteristics, or some kind of stereoscopic “placebo effect” perhaps caused by the unfamiliar experience of binocular rivalry for the interocular differences that were present. While the controversy surrounding the “Chimenti” images continued until 1864 (see Wade, 2003, for a comprehensive account), to date no studies have investigated the stereoscopic depth perception of naïve observers when viewing the original ink-wash images and the woodcut copies, and controls for demand characteristics are rare (although, see Emerson, 1864a, 1864b, 1864c, 1864d).

**Figure 8.**
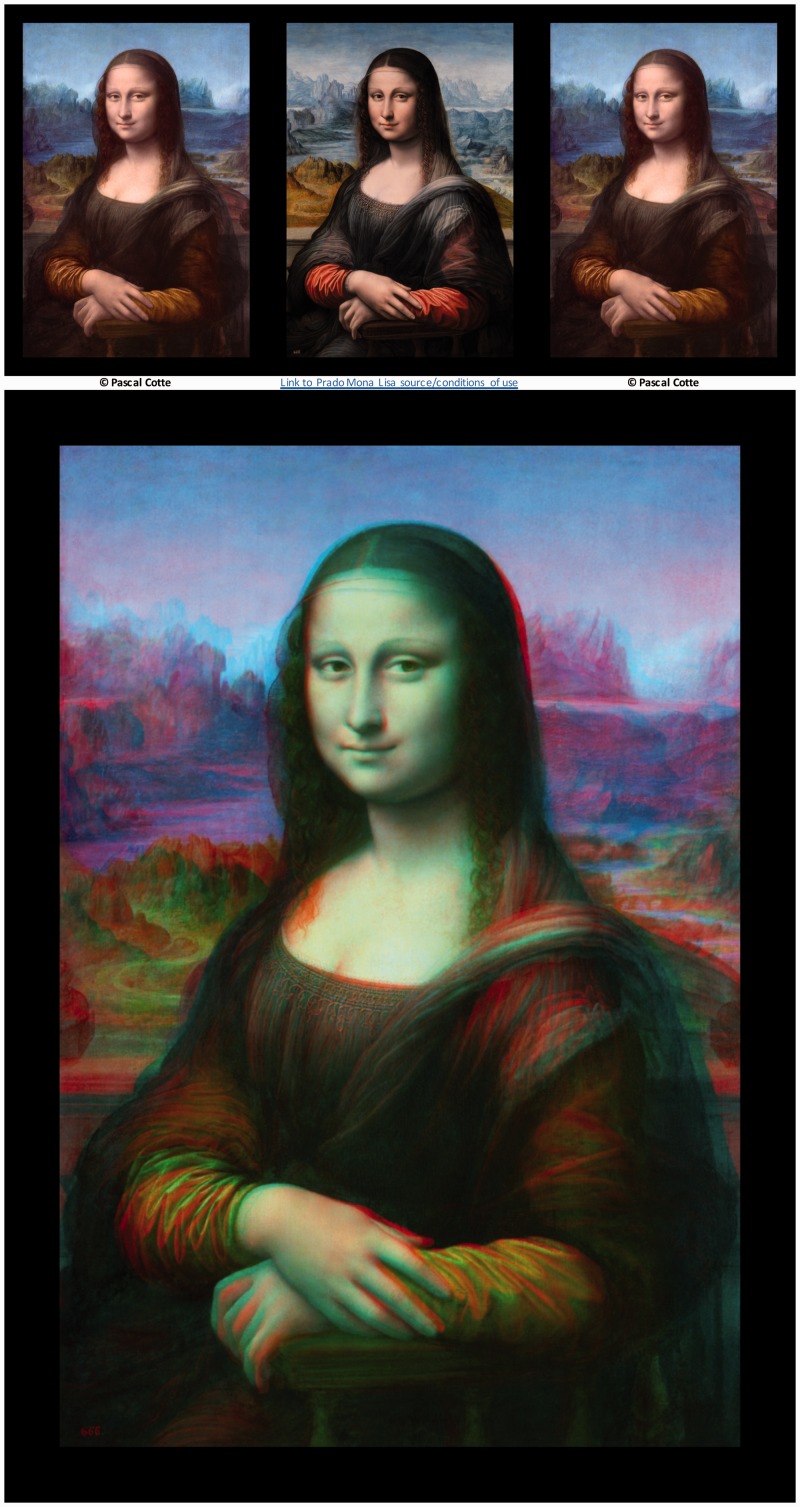
The *Mona Lisa*, presented in Universal Freeview (L-R-L) format at the top, and Dubois anaglyph format below. (a) The Prado version, believed to have been produced by an apprentice alongside Leonardo da Vinci around 1603 is presented as the left eye’s half-image. The “Louvre” version after digital restoration by Pascal Cotte (Elias & Cotte, 2008) who retains all copyright is presented as the right half-image. Although the original Louvre version was also used in this study, the restored version offers an enhanced chance of stereoscopic depth due to the greater similarity of colour palette.

The claim that Leonardo’s Mona Lisa was the first stereogram was recently advanced by Carbon and Hesslinger (2013, 2015), after scans on a version in the Prado museum (see Figure 8) revealed underlying preparatory sketches and layers of paint corrections that appeared highly similar to those on the version in the Louvre Museum in Paris, painted by Leonardo da Vinci between 1503 and 1506. This led the authors to believe that the Prado version was produced by an apprentice working alongside Leonardo, resulting in two images of the subject from slightly different viewpoints. The differences between the two perspectives were confirmed both by subjective human judgements and objective analysis by computer modelling. However, these differences were often vertical as well as horizontal (see Carbon & Hesslinger, 2013; Figure 1(c)); a feature of the results that appears incompatible with the suggestion of a stereopair. Nevertheless, the authors assert that these images “can be combined to an image of Mona Lisa that has obvious stereoscopic qualities.” The authors in particular highlighted the hands and face as areas in which the “changed perspective can most easily be observed,” and hence these areas should show particularly strong stereoscopic cues. While stereoscopic versions (with colours altered to match more closely) were presented (both as anaglyphs, and as side-by-side images for parallel free fusion), no data on perceived depth were provided.

### The Current Study

#### Artistic works under investigation

This study evaluates the quality of the stereoscopic depth effects produced by the original Wheatstone Arch images, the Chimenti image, and the *Mona Lisa* images, along with stereoscopic paintings produced by the contemporary artist Salvador Dalí. These include *The Chair, Gala’s Christ, Las Meninas, The Wash Basin, Hand Drawing Back, Painting Gala from the Back*, and *Gala’s Foot*, which can be seen in stereoscopic format in (Descharnes & Néret, 2013). The work of post-Wheatstone artists such as Dalí is informative in this context as it may help us to establish the limits of what is realistic or feasible (in terms of the production of compelling stereoscopic depth) in artistic works when the creator has full knowledge of the principles of binocular disparity. We use both versions of the Chimenti images (ink-wash and woodcut) and various combinations of *Mona Lisa* images. In addition to the basic pairing of the Prado and the original Louvre versions, subjects also rated rectangular sections of this binocular image showing only the hands, or the face. In addition, due to the vast differences in colour between the Prado version, which was restored in 2012, and the Louvre version, whose colours have faded over the centuries, we added a combination of the Prado and a digitally restored version of the *Mona Lisa* (Elias & Cotte, 2008), kindly provided by the authors. Stereoscopic effects are hypothesised for all of these image pairs. We also used a third version of the *Mona Lisa* painted by Tor Egil Hansen in 2005. This version will inevitably include some differences from the original Louvre version and may produce a degree of binocular rivalry, but as a direct copy (rather than a version of the subject produced from a different viewpoint), this binocular pair should not be expected to generate any stereoscopic depth.

#### Maximizing the chances of stereoscopic depth

The effective simulation of stereoscopic depth is dependent on many factors including the appropriate matching of the two monocular half-images. Incorrect matches may serve to degrade stereoscopic cues when they are present but may also introduce disparities in binocular images that, in reality, have none. For example, an incorrect rotational alignment may reduce the ease with which a genuinely stereoscopic image can be fused and greatly degrade the quality of the depth percept. However, the same misalignment would introduce a depth gradient in a binocular image of a flat vertical line, for example. Similar problems can be encountered with differences in shear or image width (differences in aspect ratio). Although stereoscopic photographs can be aligned using automatic image matching software, correcting for problematic differences in half-image size, rotation, and position, this process is less reliable for *sfumato* style artistic works such as the *Mona Lisa*, which lack clear edges. For this reason, where necessary, stereoscopic alignment was performed manually by stereophotographers with decades of experience of stereoscopic alignment, allowing adjustments of relative size, vertical and horizontal position, and rotation only.

In addition, even when they are geometrically appropriate, the inclusion of very large disparities leads to diplopia (double vision) and an impoverished depth percept, in addition to feelings of discomfort while viewing. While vision scientists advise that such problems can be avoided when disparities are within Panum’s fusional area, stereophotographers refer to the “1 in 30” rule, suggesting that disparities smaller than 1/30th of the total image width can usually be fused without substantial discomfort (Herbig, 1995). In this study, we were keen to ensure that all images included horizontal disparities that were smaller than either of these values, ensuring maximal viewing comfort and favourable conditions for the appreciation of stereoscopic depth.

#### Control for nonstereoscopic effects

In evaluating the stereoscopic effects, it is essential to control for the influence of monocular depth cues such as occlusion, shadow, and perspective mentioned earlier (and any other monocular sources of depth information), which are expected to be present. In addition, when assessing subjective reports of perceived depth, it is important to control for the possibility of placebo effects. It is entirely possible that the experience of binocular differences unrelated to depth may produce an unusual percept that may cause an observer to give unreliable reports of exaggerated depth. In addition, it is possible that depth effects may be gleaned from binocular rivalry without disparity, as in the *rivaldepth* (O’Shea & Blake, 1987) or the *sieve effect* (Howard, 1995) phenomena, due to differences in the colour or luminance between the two eyes. For these reasons, it is not appropriate to use the monocular half-images (whether presented to one eye alone, or whether the same image is simultaneously presented to both eyes, as in a synoptic image) as control stimuli.^1^

Effective stereoscopic art—especially works depicting recognisable real-world (as opposed to abstract) scenes—would be expected to include both monocular and binocular depth cues. While monocular cues are effective regardless of how many eyes are being used for viewing, or which eye it is that does the viewing, the same cannot be said for stereoscopic depth cues. For stereoscopic images, binocular viewing is essential, and the stereo half-image that is seen by the left versus the right eyes is crucial. In the appropriate configuration, the pattern of binocular disparities in the images is consistent with those seen in real-world viewing, and convincing depth is effortlessly perceived. However, if the two eyes’ half-images are swapped, in what is referred to as *pseudoscopic* format, the pattern of binocular disparities is reversed, so that binocular features that previously specified a near depth now specify a far depth and vice versa. The binocular geometry of the stimulus has effectively been turned “inside out.” For abstract patterns lacking monocular depth cues, such as random dot stereograms, pseudoscopic presentation simply results in a percept that is equal and opposite to the stereoscopic situation, but when monocular cues are included, such as in stereoscopic photographs or artwork, the situation is more complex. While the patterns of binocular disparities specify a world turned inside out, monocular cues such as occlusion, shadow, and perspective continue to specify the same depth as in the stereoscopic situation. This produces cue conflict: a perceptual battle between two competing sets of depth signals that results in an impression of reduced (rather than inverted) depth, accompanied by feelings of visual discomfort and percepts of incoherent depth (Jastrow, 1900; Zajac, 1964), presumably due to differences in relative strength of monocular and binocular cues in different image regions. Assuming that there are significant binocular disparity signals in a given pair of stereoscopic half-images, this allows us to make very different predictions of perceived depth for stereoscopic versus pseudoscopic stimuli. While the monocular and binocular cues should be concordant for stereoscopic presentations, producing vivid and coherent perceived depth, for pseudoscopic situations the cues will be discordant, resulting in an incoherent and reduced-depth percept. Here, a difference between depth ratings in the stereoscopic versus pseudoscopic conditions reveals the presence of significant binocular disparity cues. However, if binocular disparity signals are absent, then only monocular cues remain. Given that these cues are unchanged by reversal, in such cases the same depth and coherence ratings would be predicted for stereoscopic and pseudoscopic images. Hence, in this study, the dependent variable of interest is the *difference* in ratings between stereoscopic and pseudoscopic presentations. Given the likely presence of monocular depth cues in each image, and that they are preserved regardless of which eye views the left or right half-image, differences between stereoscopic and pseudoscopic ratings are not expected to approach the maximal values. Nevertheless, any score significantly different from zero would denote a genuine stereoscopic contribution.

## Methods

### Subjects

Participants were 12 naïve observers (7 female) with stereoacuity of 40 arcmin or better, as established by the Stereo Fly test (Stereo Optical Co., Inc., IL). Participants wore their best optical corrections beneath their stereoscopic filter glasses where necessary. Each gave informed consent before testing commenced.

### Apparatus

**Figure 9.**
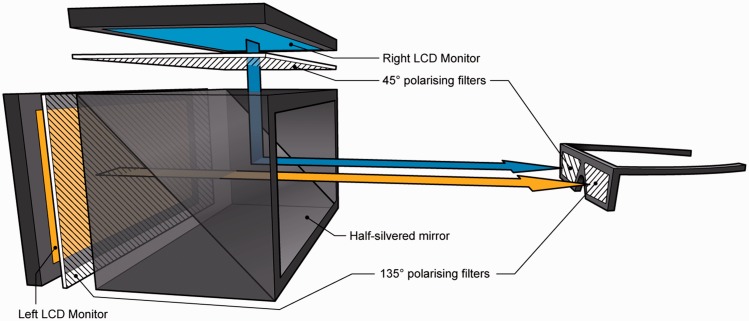
Schematic diagram of display apparatus: The True3Di SDM-240 stereomonitor.

All stimuli were displayed on a True3Di SDM-240 24″ stereoscopic 3D monitor, featuring a half-silvered mirror positioned at 45° between two perpendicularly positioned, linearly polarised liquid-crystal display panels (see Figure 9). While detailed measurements of cross talk were not taken, monocular inspection through the polarising glasses revealed no visible trace of the contralateral image. Each had a resolution of 1920 × 1200 pixels and was frame-synchronised at 60 Hz. The experiment was conducted on a PC running Windows XP 32-bit with an Intel Core i7-980X 3.33 GHz processor, 8 GB RAM, and NVIDIA Quadro FX 4000 graphics card. The subject’s viewing position was level with the middle of the screen and 2.95 m from the screen surfaces. From this distance, the 0.5184 × 0.324 m screen subtended a visual angle of 10.0 × 6.3°, with each pixel subtending 0.31 × 0.31 arcmin.

### Stimuli

**Figure 10.**
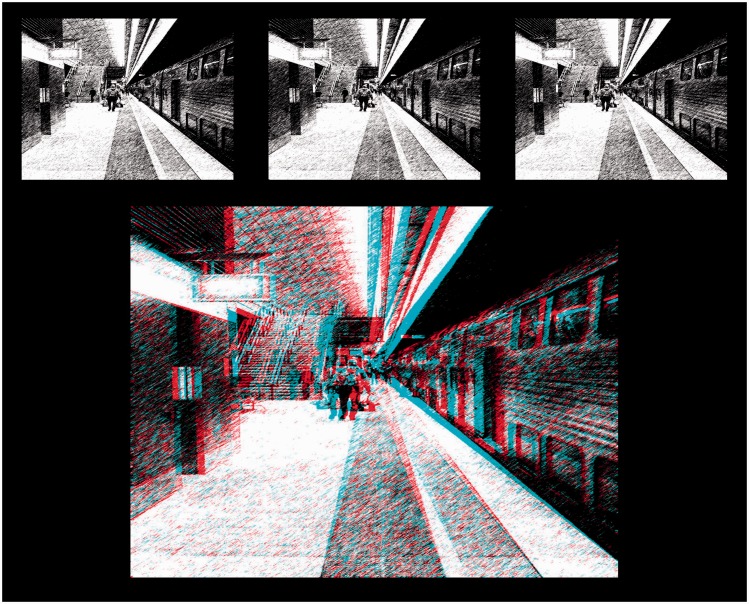
Paradigm check image #2 (Train), presented in Universal Freeview (L-R-L) format at the top, and Dubois anaglyph format below.

**Figure 11.**
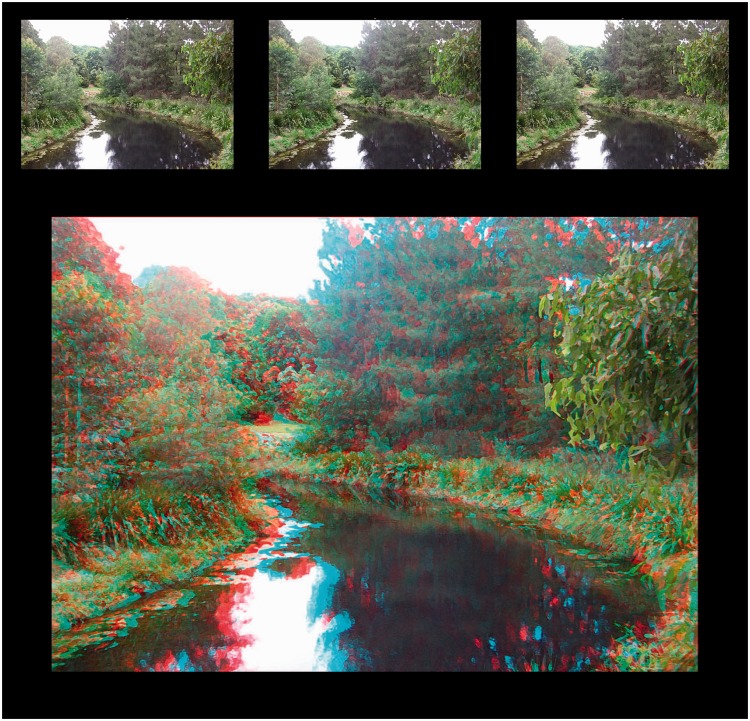
Paradigm check image #3 (River), presented in Universal Freeview (L-R-L) format at the top, and Dubois anaglyph format below.

Pairs of half-images from potential stereoscopic art works were selected from various online resources and downloaded at the highest available resolution. In addition, three stereoscopic photographs taken by the author were transformed by Photoshop’s Artistic or Sketch filters (dry brush, watercolour, graphic pen). Given that their disparities were geometrically appropriate and consistent throughout, these images were intended to represent something of a “best case scenario” and served as paradigm check stimuli. Two examples of these images are shown in Figure 10 (Train) and Figure 11 (River).

Stimulus alignment was performed using automatic processes in stereoscopic photo manipulation software (StereoPhoto Maker) wherever possible, using adjustments of relative image size, horizontal and vertical position, and rotation only. Where such attempts failed, experienced stereophotographers who were naïve as to the study’s hypotheses were recruited to perform precise stereoscopic adjustments using the same set of transformations. Lastly, the size of each stereoscopic half-image was adjusted (without affecting their relative alignment) to ensure that none of the pairs involved disparities that exceeded Panum’s fusional area (around 18–20 arcmin; Grove & Harrold, 2013; Schor & Tyler, 1981), which, for the current display apparatus and viewing distance, matches the maximum disparity based on the 1/30 rule. Onscreen parallax was never greater than 1.5 cm, giving a maximum disparity of 17 arcmin, and a maximum disparity-screen ratio of 1:35. During debriefing, no participants mentioned any experience of diplopia. Images were presented with a black surround (as shown in Figures 6 to 8, 10, and 11), which filled the remainder of the screen.

### Procedure

Before data collection, participants were familiarised with the display and apparatus by viewing some examples of stereoscopic art in which one of the monocular views had been created by copying the original image and introducing disparities in certain areas of the image, along with some stereoscopic photographs that had been processed in Adobe Photoshop to simulate an artistic appearance in the same way as the paradigm check images above. Pseudoscopic images were also shown to the observers to familiarise them with the reduced and incoherent depth, and the slight visual discomfort that these images can produce. During this phase, participants were encouraged to scrutinise the image and describe their depth percepts to the experimenter.

A short practice session followed, involving five stereoscopic images not included in the main experiment, and their pseudoscopic equivalents. In this session, participants followed the same procedure as the experiment proper. The order of image presentation was randomised for each participant. Each trial involved the presentation of a half-image pair for 10 s during which the participant was instructed to inspect the image binocularly. After this 10 s period, the images remained on screen while the participant made two ratings in their own time. The first rating was of the degree of depth within the image, as an integer from 0 to 10 inclusive. To allow comparison of images in which the magnitude of simulated depth can vary extensively, participants were encouraged to give a high rating if the depth appeared compelling and appropriate to the scene, not just if the depth appeared extensive. Next, participants were asked to make a rating of depth coherence, considering whether there is consistency between the apparent depths of various aspects of the image (i.e., do they appear in the expected order, or does the depth of the image appear “messy”?). In particular, when rating the Mona Lisa images, participants were asked to make these judgements concerning the depth in the main subject herself, ignoring any percept of depth between the image and the background.^2^

### Data Analysis

For each of images under investigation, depth and coherence ratings were considered separately. Data are presented in terms of the difference between ratings for stereoscopic and pseudoscopic versions, averaged across observers. Where 95% confidence intervals overlap zero, this indicates a lack of a significant difference between the two, and hence a lack of evidence of stereoscopic depth. In addition, the results of paired *t* tests between stereoscopic and pseudoscopic scores are reported.

## Results and Discussion

### Paradigm Check and Wheatstone Arch Stereograms (1838)

**Figure 12.**
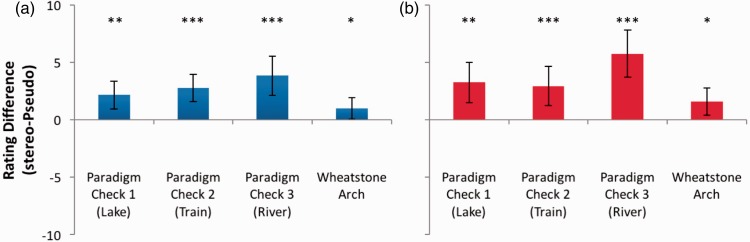
(a) Depth and (b) coherence rating differences for artistically filtered stereoscopic paradigm check photographs (Lake, Train, and River) and the Wheatstone Arch. Error bars represent 95% confidence intervals. *p ≤ .05; **p ≤ .01; ***p ≤ .001.

To validate the logic and procedure of the study, subjects rated geometrically consistent stereoscopic photographs that had been manipulated using Photoshop to simulate artistic brush or pen strokes. Figure 12 demonstrates the convincing depth (Lake: *t*(11) = 3.952, *p* = .002; Train: *t*(11) = 5.109, *p* < .0005; River: *t*(11) = 4.934, *p* < .0005) and coherence (Lake: *t*(11) = 4.068, *p* = .002; Train: *t*(11) = 3.772, *p* = .003; River: *t*(11) = 6.127, *p* < .0005) conveyed by the paradigm check images. These results confirm the effective simulation of depth in our binocular set up and confirm the utility of our experimental paradigm in establishing the presence of genuine and coherent stereoscopic depth when it is present.

In addition, significant stereoscopic depth, *t*(11) = 2.345, *p* = .039, and coherence, *t*(11) = 2.916, *p* = .014, were demonstrated for the Wheatstone Arch. Although significant, these effects were hardly impressive. On closer inspection of the half-images, it becomes clear that a geometrical inconsistency is present. While the bottom section of the structure (both left and right sides) shows considerable relative disparity between the front and rear corners, the central upper portion features a smaller relative disparity, despite these two sections appearing continuous in each monocular half-image (see Figure 6 anaglyph). Given Wheatstone’s clear appreciation of the principles of binocular disparity, this may reflect his considerably more modest skills as an artist^3^ than as a scientist.

### Chimenti Images

**Figure 13.**
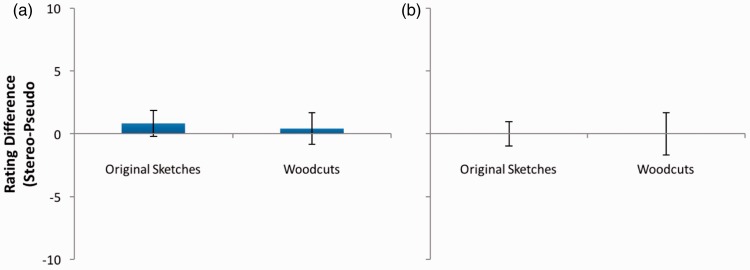
(a) Depth and (b) coherence rating differences for the Chimenti images. Error bars represent 95% confidence intervals.

Since 1860, claims that images produced by Jacopo “Chimenti” da Empoli include clear stereoscopic depth have been made by various individuals. However, as shown in Figure 13, these images showed no significant depth or coherence effects (*p* > .05 in all cases). While extending their investigations by assessing both the woodcuts and original sketches, these data agree with the measurements of Emerson (1864d) and the conclusions of Joy (1864) and Wade (2003), allowing us to reject the assertions of Brewster (1860) that the world’s first stereoscopic images were drawn by Jacopo da Empoli.

### Mona Lisa Images

**Figure 14.**
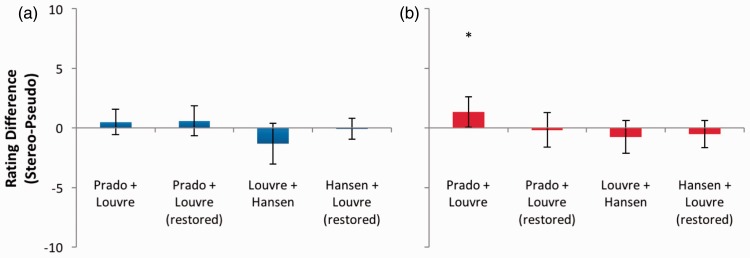
(a) Depth and (b) coherence rating differences for full portrait images of the Mona Lisa. Error bars represent 95% confidence intervals.

Recent claims that Leonardo da Vinci’s *Mona Lisa* produces a convincing impression of stereoscopic depth were tested using a number of different combinations and regions of the image, to attempt to provide the best chance of identifying any such effects. Figure 14 displays the results for full Mona Lisa images. Considering stereopairs for which Carbon and Hesslinger (2013, 2015) encourage us to hypothesise stereoscopic effects (Prado-Louvre versions), differences between depth ratings in stereo and pseudo mode were small, with confidence intervals substantially overlapping zero both for depth and coherence (*p* > .05 in all cases), except for in one case. For the combination of the Prado and the original (unrestored) Louvre half-images, the mean difference between coherence scores for stereoscopic and pseudoscopic presentations was positive, producing a just-significant result at the *p* = .05 level, *t*(11) = 2.292, *p* = .043. As expected, effects of depth and coherence were absent for the Hansen versions (*p* > .05 in all cases).

**Figure 15.**
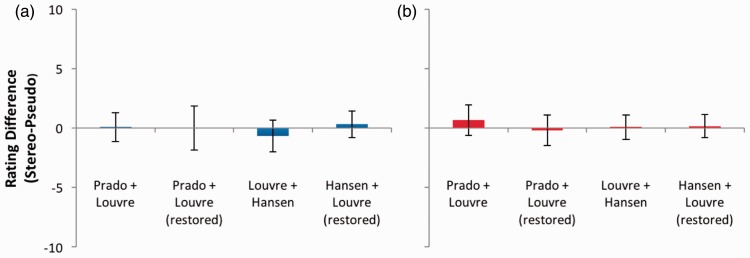
(a) Depth and (b) coherence rating differences for images of Mona Lisa’s head. Error bars represent 95% confidence intervals.

**Figure 16.**
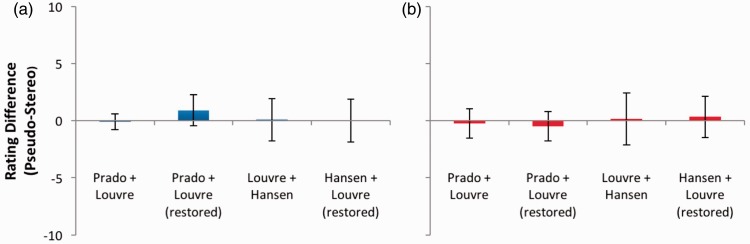
(a) Depth and (b) coherence rating differences for images of Mona Lisa’s hands. Error bars represent 95% confidence intervals.

For isolated images of the head (Figure 15) and the hands (Figure 16)—two areas of the image that Carbon and Hesslinger (2013) highlight as being particularly rich in stereoscopic cues to depth—no significant effects of depth or coherence were present in any image pair (*p* > .05 in all cases).

In sum, contrary to the claims of Carbon and Hesslinger (2013, 2015), there is no evidence of stereoscopic depth in any version of the Mona Lisa figures. The single marginally significant result identified here refers to the coherence of perceived depth in an image *lacking stereoscopic depth*. As such this result is most sensibly interpreted as a Type I error.

### Dalí Images

At this stage, the results may appear underwhelming, given that so few of the images appear to contain significant stereoscopic structure. It is noteworthy that, of the images tested, only the paradigm check images (created from relatively objective stereoscopic photographs, featuring consistent geometry) and the Wheatstone Arch (a simple line drawing which appears to have been constructed with the aid of a straight edge) have proven to be effective. When significant artistic influence is involved, as in the sketches of Chimenti or the paintings of Leonardo (plus apprentice?), stereoscopic effects are absent. Given that stereoscopic images often involve quite small disparities that might approach the artist’s limits of precision with a paintbrush (or other artistic device), one might be forgiven for wondering whether the demands of stereoscopic painting are simply too great, or that the procedure used here is not sufficiently sensitive to detect the subtle stereoscopic effects. These observations necessitate the inclusion of images produced after the depth-enhancing effects of stereoscopic vision had become widely known (i.e., after Wheatstone’s (1838) seminal work, the rise in popularity of stereophotography (Wade, 2012), and the 3D movie “boom” of the 1950s (Zone, 2005)). Is stereoscopic painting even possible?

**Figure 17.**
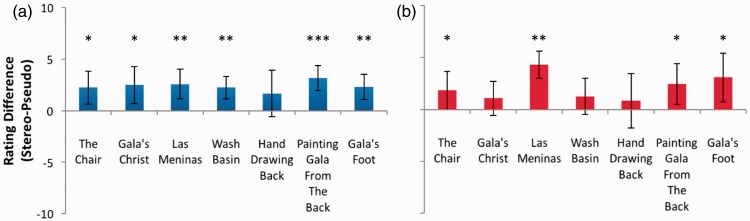
(a) Depth and (b) coherence rating differences for paintings by Salvador Dalí. Error bars represent 95% confidence intervals. *p ≤ .05; **p ≤ .01; ***p ≤ .001.

Figure 17 encourages a resoundingly positive answer. While the attempt to produce clear and convincing stereoscopic depth is not supported by significant results for *Hand Drawing* Back^4^ (*p* > .05 for depth and coherence), this may be unsurprising. Here, Dalí has deliberately given the principal subject a stereoscopic depth beyond that of the sun, even though occlusion specifies the opposite arrangement. Even if the artist achieved exactly what was intended with this image, this binocular or monocular cue conflict would be predicted to produce little or no difference in ratings between stereo and pseudo versions. On the other hand, significant depth effects were shown for all of the other Dalí stereograms that were tested; *The Chair*: *t*(11) = 3.129, *p* = .010*; *Gala’s Christ*: *t*(11) = 3.079, *p* = .010*; *Las Meninas*: *t*(11) = 3.934, *p* = .002**; *Wash Basin*: *t*(11) = 4.552, *p* = .001***; *Painting Gala from the Back*^5^: *t*(10) = 5.590, *p* < .0005***; *Gala’s Foot*: *t*(11) = 4.204, *p* = .001***. While the depth was also coherent for four of these works; The Chair: *t*(11) = 2.200, *p* = .05*; Las Meninas: *t*(11) = 7.288, *p* < .0005***; *Painting Gala from the Back*: *t*(10) = 2.645, *p* = .025*; *Gala’s Foot*: *t*(11) = 2.903, *p* = .014*, this was not significant for the other two (*p* > .05 in all cases. These failures may again be the result of Dali’s presumably deliberate inclusion of cue conflict in each of these works.

## Conclusion

The current study aimed to investigate, using rigorous experimental methods, claims that Jacopo “Chimenti” da Empoli and Leonardo da Vinci produced stereoscopic images predating those presented by Wheatstone in 1838. Although it is impossible for an experimental study to establish the intention of the artists in question, it is possible to evaluate the effects caused by viewing their creations. While no images by these artists produced evidence of stereoscopic structure, clear binocular depth was evident for paintings by Salvador Dalí that were produced after the details of stereopsis has been widely disseminated. In light of these results, the first stereoscopic images appear to be Sir Charles Wheatstone’s rudimentary line drawings, including the Wheatstone Arch (Wheatstone, 1838).

For the Chimenti images, this result may not be surprising. Despite adamant claims by Brewster (1860), several previous authors have attested that coherent stereoscopic relief is absent in these images (Emerson, 1864a, 1864b, 1864c, 1864d; Joy, 1864) as summarised by Wade (2003). In addition, no corroborating evidence, such as writings or other attempts to produce stereoscopic depth, can be found to suggest that Jacopo understood the geometric principles of binocular depth perception. However, the same cannot be said for Leonardo. In his *Trattato della Pittura* (A Treatise on Painting), which includes observations believed to have been made between 1492 and 1513 (Pedretti, 1965), Leonardo observed that when viewing real-3D scenes, the two eyes see subtly different images. However, rather than concentrating on the binocular disparities (i.e., the differences in the positions of binocularly visible objects) that constitute the major binocular cue to depth, he observed and documented the circumstances under which some visual features may be visible to one eye, yet occluded in the other. This is the binocular depth cue of half-occlusion, sometimes referred to as da Vinci Stereopsis (Brooks & Gillam, 2007; Cook & Gillam, 2004; Nakayama & Shimojo, 1990). In addition, Leonardo expressed his frustration that a flat painting could never fully recreate the depth that is experienced when viewing real objects, unless it is viewed with one eye (Wade, Ono, & Lillakas, 2001). In this context, it is certainly a seductive idea that the *Gioconda*—perhaps the most celebrated artwork in history—was actually Leonardo’s attempt to address this problem and create a more satisfying pictorial representation of reality. However, it seems doubtful that the polymath would have chosen a live, inconstant subject for any such experiment, in preference to drawing a still life scene that could be reproduced more reliably and whose half-images would be more likely to match appropriately. Or, for that matter, that the two views would have been produced by two different artists (apparently himself plus an apprentice) with differing styles, rather than *The Renaissance Man* himself producing both views. Although there is clearly no evidence of stereoscopic depth in the images under investigation here, it remains possible that such well-matched stereoscopic works of Leonardo da Vinci lie undiscovered in a dark corner of a museum archive, awaiting discovery.

## Notes

1 In addition, this situation would produce an invalid comparison between images that would not be matched for Fourier properties or for colour content regardless of which of the monocular images is chosen to create the synoptic image.

2 This feature of the Mona Lisa has been the subject of considerable controversy, with many art historians disputing the location while others claim that it represents a flat backdrop that the model posed before, or that it was conjured purely by the artist’s imagination (Carbon & Hesslinger, 2015; Smith, 1985). As such it may be unrealistic to expect this aspect of the image to include stereoscopic depth cues.

3 The possibility that Wheatstone commissioned another individual, such as an engraver, to produce these images, should be noted. However, any such individual is likely to have been under strict direction from the author, and as such Wheatstone is regarded as the producer of these images.

4 Full title: *Hand Drawing Back the Golden Fleece in the Form of a Cloud to Show Gala, Completely Nude, the Dawn, Very, Very Far Away Behind the Sun* (1977).

5 Full title: DalιL Seen from the Back Painting Gala from the Back Eternalized by Six Virtual Corneas Provisionally Reflected by Six Real Mirrors (1972–1973). Due to a program error, data from one participant were not recorded for this image.
